# The Effect of Metformin on Self-Selected Exercise Intensity in Healthy, Lean Males: A Randomized, Crossover, Counterbalanced Trial

**DOI:** 10.3389/fendo.2021.599164

**Published:** 2021-02-25

**Authors:** Nanna Skytt Pilmark, Christina Petersen-Bønding, Nielse Frederich Rose Holm, Mette Yun Johansen, Bente Klarlund Pedersen, Katrine Bagge Hansen, Kristian Karstoft

**Affiliations:** ^1^ Centre for Physical Activity Research (CFAS), University of Copenhagen, Rigshospitalet, Copenhagen, Denmark; ^2^ Steno Diabetes Center Copenhagen, Gentofte, Denmark; ^3^ Department of Clinical Pharmacology, Bispebjerg Hospital, University of Copenhagen, Copenhagen, Denmark

**Keywords:** exercise, metformin, rate of perceived exertion, type 2 diabetes, self-selected exercise intensity

## Abstract

**Introduction:**

In general, patients with type 2 diabetes have lower cardiorespiratory fitness levels and perform exercise at lower intensities compared to healthy controls. Since metformin (MET) has been shown to increase the rate of perceived exertion (RPE) during exercise with a fixed intensity, MET *per se* may reduce self-selected exercise intensity. The aim of this study was to assess the effect of MET on self-selected exercise intensity.

**Methods:**

Healthy males were eligible for this crossover, counterbalanced study with two treatment periods: MET and placebo (PLA), each lasting 17 days. Treatment dose was gradually increased and reached 2 g/day on treatment day 9, and continued at that level for the rest of the treatment period. The two periods were performed in randomized order. Two experimental days (A+B) were conducted on Day 15 (A) and Day 17 (B) of each period, respectively. Day A consisted of an exercise bout with self-selected exercise intensity (equal to RPE = 14–15 on the Borg Scale). Day B consisted of an exercise bout with fixed intensity (70% of VO_2_peak). Oxygen consumption rate was assessed continuously during both exercise bouts.

**Results:**

Fifteen males (age 23.7 ± 0.6 years, BMI 22.3 ± 2.0, VO_2peak_ 3.5 ± 0.6 L/min) were included in the study. On Day B, RPE was higher in MET compared to PLA (14.8 ± 0.4 vs. 14.0 ± 0.3, *P* = 0.045). On Day A, no difference in self-selected exercise intensity measured by oxygen consumption rate (PLA 2.33 ± 0.09 L O_2_/min, MET 2.42 ± 0.10 L O_2_/min, *P* = 0.09) was seen between treatment periods.

**Conclusions:**

Self-selected exercise intensity was not reduced by MET in healthy males, despite the fact that MET increased RPE during an exercise bout with fixed intensity.

## Introduction

Physical activity is a cornerstone in the treatment of patients with type 2 diabetes (1). However, most patients with type 2 diabetes are not able to achieve satisfying glycemic control with physical activity alone, which is why pharmacological treatment is often initiated. Metformin is the initial glucose-lowering drug of choice for patients with type 2 diabetes, and most patients are prescribed metformin as a lifelong treatment shortly after the diagnosis (2). Compared to matched, healthy controls, patients with type 2 diabetes have lower cardiorespiratory fitness levels (3) and, perform free-living, unsupervised exercise at lower intensities (4); intensities that are too low to induce robust metabolic improvements (5). The reason for this unclear, but reduced mitochondrial function in skeletal muscle has been suggested as a potential explanation (6). Moreover, and potentially in continuation of this, the rate of perceived exertion (RPE) may play an important role, since patients with type 2 diabetes are known to report higher RPE during exercise with a given intensity, compared to healthy controls (7). Although debatable (8), it has been reported that RPE is associated with blood lactate levels (9), and since metformin treatment has been shown to increase blood lactate levels, heart rate (HR) and RPE in healthy individuals at a given exercise intensity (10), it may be speculated that it is the metformin treatment *per se* and not the diabetes phenotype, which is responsible for the low self-selected exercise intensity in patients with type 2 diabetes. Therefore, the aim of this study was to assess the effect of metformin on self-selected exercise intensity. We hypothesized that metformin, possibly through an increase in blood lactate and heart rate, would increase RPE, and thereby decrease the self-selected exercise intensity. We chose to test this hypothesis in young, healthy, lean males in order to reduce variance due to heterogeneity in the included population and in order to evaluate the effects of metformin treatment *per se*, independent of the diabetes phenotype and prior metformin treatment.

## Methods

Healthy, lean (BMI<25), low-to moderately physically active (≤150 min of structured physical activity/week), male individuals were eligible for the study. Exclusion criteria were: smoking, daily medication, contraindications to increased levels of physical activity (11), liver cell damage (ALT/AST at least 3 times above upper normal level), prior history of lactic acidosis and eGFR<60 ml/min.

To ensure that all inclusion and no exclusion criteria were met, a screening day was performed. The screening day included a medical examination, recording of medical history, a blood chemistry screen, an ECG and a cardiorespiratory fitness (VO_2_peak) test on a cycle ergometer (Monark 739E, Varberg, Sweden) using indirect calorimetry (Cosmed Quark, Rome, Italy). The VO_2_peak test started with a 5-min warm up at 80 Watt (W), after which the load was gradually increased by 20 W every minute until at least one of the following criteria was met: plateau of HR and VO_2_ with incremental workloads, respiratory exchange ratio > 1.1, or volitional exhaustion, as previously described (12). Written informed consent was obtained from all participants. The study was approved by the ethical committee of the Capital Region of Denmark (H-16032037) and registered at ClinicalTrials.org (NCT02951260).

### Study Design and Treatment Periods

Participants fulfilling inclusion criteria were included in a double-blinded, crossover, counterbalanced study with two treatment periods performed in a randomized order. The two treatment periods were identical except for the following treatment:

PLA: Placebo treatment (17 days)
MET: Metformin treatment (17 days)

To ensure adherence to the treatment protocol and maintain participant blinding, both metformin (MET) and placebo (PLA) treatments were gradually increased to minimize gastrointestinal discomfort caused by metformin.

- Treatment days 1–4: 500 mg × 2
- Treatment days 4–8: 500 mg + 1000 mg
- Treatment days 9–17: 1000 mg × 2

After completion of the first treatment period (first 17 days of treatment), a 4-day washout period was applied before initiation of the second treatment period.

**Figure 1:**
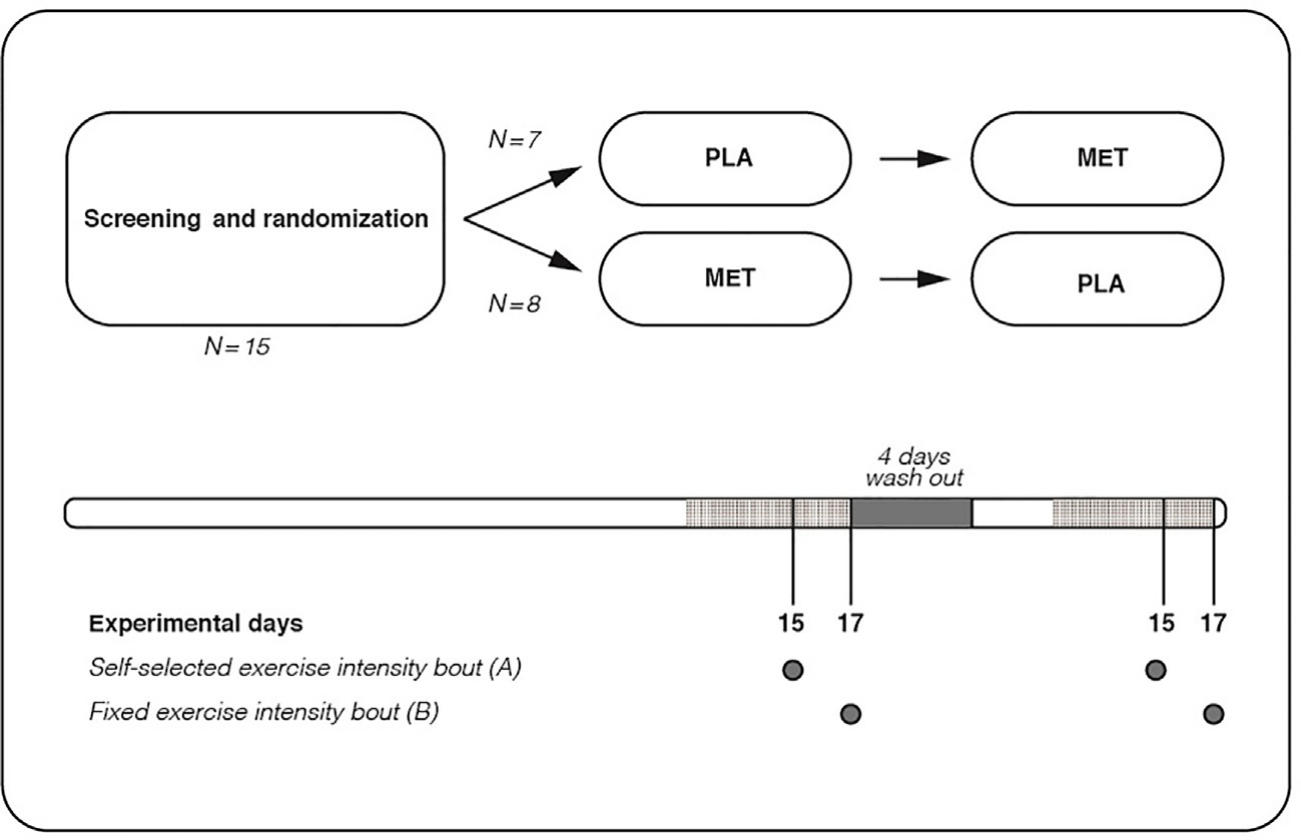
Study design. Shaded area indicates that diet records were kept from day 13 to 17 in the first treatment period, and subsequently this diet was mirrored from day 13 to 17 in the 2^nd^ treatment period.

On day 15 and 17 of each treatment period, participants underwent two experimental days (Experimental day A and B, respectively). Participants were instructed to keep a diet record from day 13 to day 17 in the first treatment period, and subsequently to follow this diet closely during the same days in the 2^nd^ treatment period. **Figure 1** shows the study design.

### Randomization and Blinding

Participants were randomly allocated, using a computer-based algorithm (randomizer.org) to one of two arms, MET or PLA, with an allocation ratio 1:1. The random number sequence was created by an endocrinologist (KBH), not involved in any of the study procedures. The sequence was stored and concealed from other staff and participants in a locked cabinet in a locked office at a geographical location different from the place of the study procedures.

All study personnel and participants were blinded to the allocation throughout the study period, until all participants had completed their last test and all outcomes were computed. The two study medications (visually identical pills) were prepared by a clinical pharmacy (Region Hovedstadens Apotek, Herlev Hospital) and delivered to KBH, who dispensed the medication in identical packages according to the allocation number. Following the allocation, KBH delivered the identical pre-packed medications to the test centre (CFAS) prior to the first test day. After the screening day, participants received the pre-packed medications with instructions on how to administer the pills and how to report serious adverse events (SAE) and adverse events (AE) to KBH. Since known side-effects of metformin, e.g., gastrointestinal pain and nausea, could potentially reveal the treatment allocation, all SAE’s and AE’s were reported to the endocrinologist, who was not involved in the study procedures, in order to maintain blinding. Moreover, the participants were instructed not to share information about any SAE’s and AE’s with the study personnel. A priori, it was decided that the concealment could be revealed upon the endocrinologist’s discretion based on the frequency and severity of SAE’s and AE’s. After completion of the 1^st^ treatment period, participants received the medication for the 2^nd^ treatment period.

### Experimental Days

Participants arrived in the laboratory 1 h after having had a standardized breakfast (60 g bun with 20 g cheese (220 kcal: fat 8.8 g, carbohydrates 24.7 g, protein 9.9 g) that was ingested with the morning dose of MET/PLA. Upon arrival, a venous line for blood sampling was placed and 1 h after arrival, a 45-min exercise bout (self-selected intensity on Experimental day A; fixed intensity on Experimental day B) was initiated, see detailed descriptions below. Prior to initiation of the exercise bout, a baseline blood sample was drawn for analyses of blood lactate and glucose (ABL 7 series; Radiometer, Denmark). These analyses were repeated every 15 min during the 45-min exercise bout and results were averaged to provide mean values during the exercise bouts.

### Self-Selected Exercise Intensity (Experimental Day A, 15 Days After Treatment Start)

On Experimental day A, participants were instructed to perform a 45-min exercise bout on an cycle ergometer equaling a subjective RPE of 14 to 15 on the Borg Scale (corresponding to “somewhat hard” or “hard”). In order to ensure maintenance of the prespecified RPE, the participants were asked every 5 min (in standardized terms) by the investigator, whether the load should be adjusted. Participants were blinded for the load, which was adjusted by 10 Watts (increased or decreased), if a change was required. In addition to the predefined, standardized 5-min intervals, participants were encouraged to ask the investigator to change the load, if needed, at any time point during the exercise bout. To ensure correct starting intensity, a 15-min warm-up period was completed prior to the actual 45-min exercise bout. During the warm-up period, the load was gradually increased, ensuring that participants would begin the exercise bout at a RPE of 5 to 15.

Three hours after termination of the exercise bout with self-selected exercise intensity, a VO_2_peak test was performed. This completed Experimental day A.

Indirect calorimetry (Cosmed Quark, Rome, Italy) and HR (Polar RS400) measurements were performed continuously both during the exercise bout and the VO_2_peak test, using a mask and breath-by-breath measurements and a heart rate strap, respectively. Oxygen consumption rate was used as a proxy measure of exercise intensity during the exercise bout with self-selected exercise intensity. Furthermore, external work (kJ) was calculated by multiplying load (Watt) by time (seconds) spent on each load, divided by 1000.

### Fixed Intensity (Experimental Day B, 17 Days After Treatment Start)

On Experimental day B, participants underwent a 45-min exercise bout with fixed intensity at 70% of VO_2_peak. The correct intensity was calculated from the VO_2_peak test performed on the screening day. The exercise bout was initiated by a 5-min warm-up period with gradually increasing intensity, ensuring that the participants would begin the exercise bout at 70% of VO_2_peak. RPE for the entire exercise bout was assessed at the termination. Indirect calorimetry and HR measurements were performed continuously during the exercise bout, analogue to Experimental day A.

### Outcomes and Sample Size

The primary outcome was differences between MET and PLA in self-selected exercise intensity, measured as oxygen consumption rate (L O_2_/min). Secondary outcomes included differences in RPE, mean blood lactate levels and HR levels during the 45-min exercise bout with fixed intensity.

No studies have, to our knowledge, investigated the effects of metformin on self-selected exercise intensity. The impact of short-term metformin treatment on RPE, blood lactate and HR has been investigated in various studies (10, 13, 14). These studies have each included between 9 and 17 participants. In general, metformin treatment has been found to robustly increase RPE, blood lactate, and HR. Based on these studies, and a pragmatic assumption that these variables would directly affect self-selected exercise intensity, we included 15 participants in the current study.

### Statistical Methods

Normality was assessed by D’Agostino-Pearson, Shapiro-Wilk, Kolmogorov-Smirnov test. All variables were normal-distributed. Therefore, between-treatment period variables were compared using Student´s paired t-test. All statistical analyses were performed by Prism version 8 (GraphPad, Canada). Statistical significance was accepted with *P*<0.05 (2-sided). Values are presented as mean ± SEM, baseline characteristics as mean ± SD.

## Results

**Figure 2:**
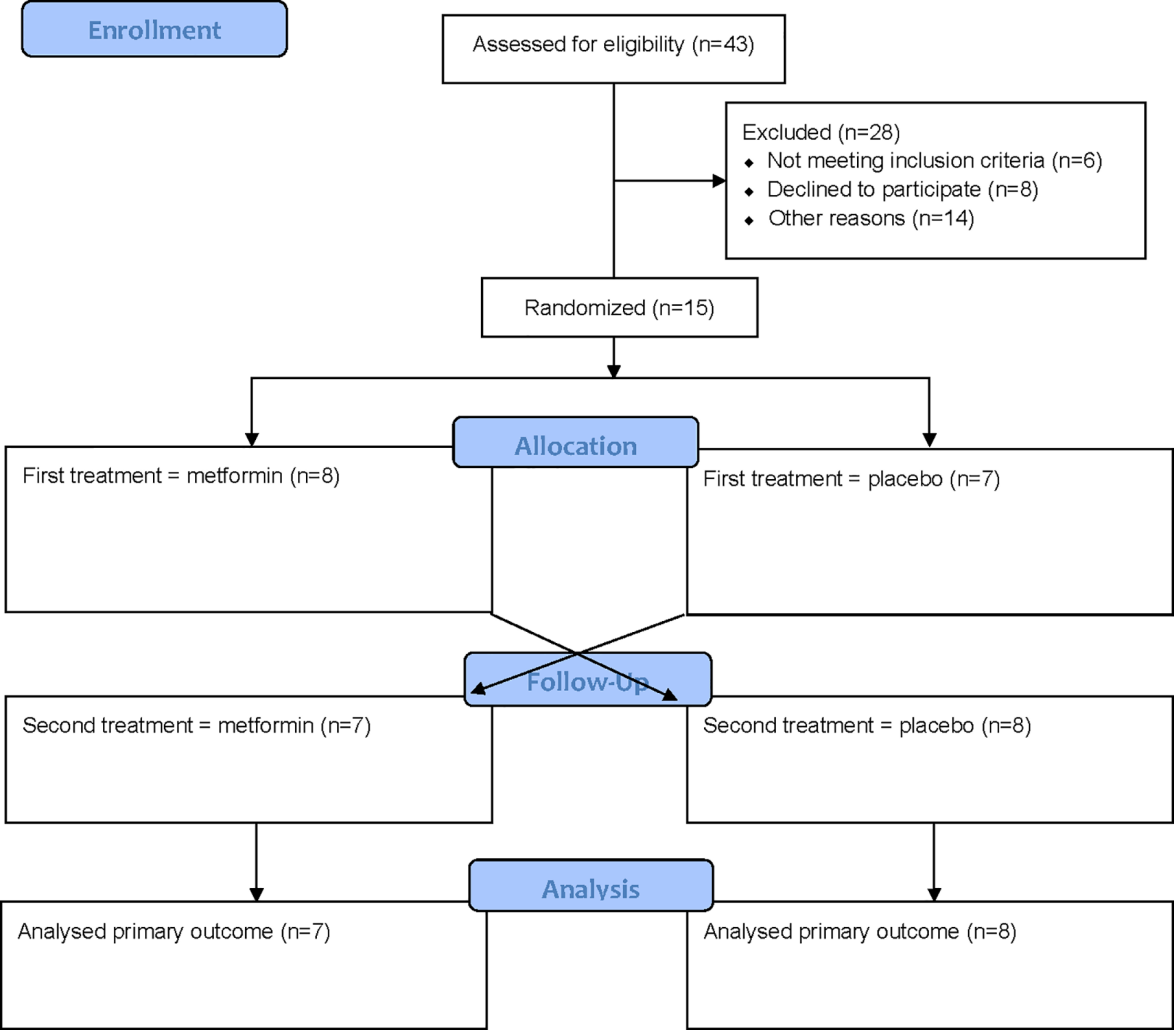
Flowchart.

Fifteen young, lean, healthy males were included and completed the study (**Table 1**). Seven individuals started with the PLA treatment period, whereas eight started with the MET treatment period (**Figure 2**). No difference in body weight was seen between treatment periods at Experimental day 17 (PLA 75.5 ± 9.8 kg, MET 75.2 ± 9.7 kg, *P* = 0.9).

**Table 1:** Baseline characteristics.

*N*	15
Age (years)	23.7 ± 0.6
Height (m)	1.8 ± 0.1
Body weight (kg)	75.3 ± 9.4
BMI (kg/m²)	22.3 ± 2.0
VO_2_peak Absolute (L/min)	3.5 ± 0.6
VO_2_peak Relative (ml/kg/min)	46.5 ± 4.9
Resting heart rate (bpm)	61 ± 8
Peak heart rate (bpm)	197 ± 9
Fasting blood lactate (mmol/L)	1.1 ± 0.4
Fasting glucose (mmol/L)	4.6 ± 0.3
HbA1c (mmol/mol)	32.7 ± 3.0

Baseline characteristics are presented as mean ± SD. BMI, body mass index; VO_2_peak, maximal oxygen consumption; HbA1c, hemoglobin A1c.

Study procedures took place from October 2016 to April 2017. All data were collected at the Centre for Physical Activity Research, Rigshospitalet, Copenhagen, Denmark.

### Exercise Bout With Fixed Intensity

RPE was significantly higher in MET compared to PLA in the exercise bout with fixed intensity (*P* = 0.045). Furthermore, a tendency towards higher blood lactate levels was seen during MET compared to PLA (*P* = 0.06). Besides that, no differences were observed between PLA and MET for any variables in the exercise bout with fixed intensity **(** **Table 2** **)**.

**Table 2:** Exercise bout with fixed intensity.

	PLA	MET	P-value
Mean oxygen consumption rate (L/min)	2.6 ± 0.1	2.6 ± 0.1	>0.9
Mean external work (kJ)	433 ± 13	442 ± 15	0.4
Mean intensity (Watt)	166 ± 5	169 ± 5	0.6
RER	0.89 ± 0.00	0.89 ± 0.01	0.8
Mean HR (bpm)	158 ± 4	164 ± 4	0.2
Percentage of HR peak (%)	86.9 ± 1.5	88.3 ± 1.4	0.4
Mean blood lactate (mmol/L)	3.0 ± 0.3	3.5 ± 0.4	0.06
Mean blood glucose (mmol/L)	4.7 ± 0.1	4.8 ± 0.1	0.4
RPE	14.0 ± 0.3	14.8 ± 0.4	0.045

All variables are presented as mean ± SEM. RER, respiratory exchange ratio; HR, heart rate; bpm, beat per minute; RPE, rate of perceived exertion. P-values were calculated from student´s paired t-test.

### Exercise Bout With Self-Selected Exercise Intensity

**Figure 3:**
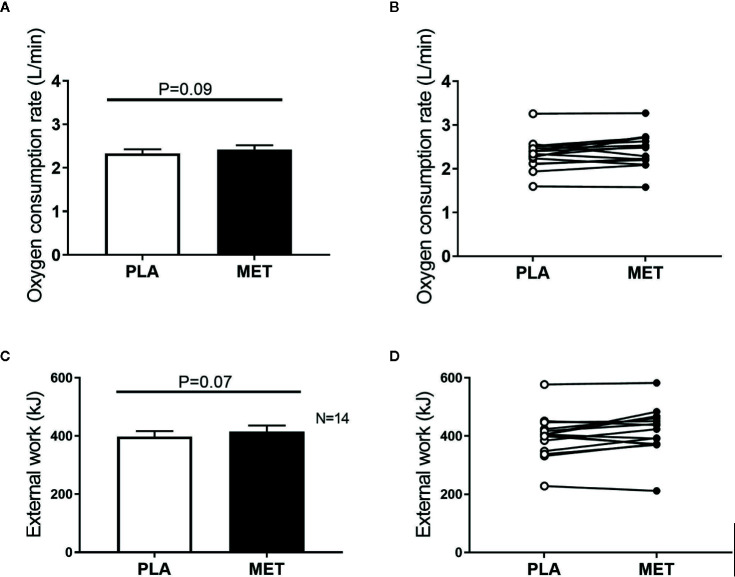
Oxygen consumption rate and external work during an exercise bout with self-selected exercise intensity. Panel **(A)** mean oxygen consumption rate (L/min) during an exercise bout with self-selected intensity. Panel **(B)** individual oxygen consumption rates (L/min) during an exercise bout with self-selected intensity. Panel **(C)** mean external work (kJ) during an exercise bout with self-selected exercise intensity. Panel **(D)** individual values of external work (kJ) during an exercise bout with self-selected exercise intensity. Values are presented as means ± SEM. P-values were calculated from student´s paired *t* test.

During the exercise bout with self-selected exercise intensity, no significant differences between treatment periods were observed in either oxygen consumption rate (PLA 2.33 ± 0.09 L O_2_/min, MET 2.42 ± 0.10 L O_2_/min, *P* = 0.09) (**Figures 3A, B** **)**, mean Watt (PLA 154 ± 6, MET 159 ± 6, *P* = 0.3), external work (PLA 398 ± 19 kJ, MET 415 ± 21 kJ, *P* = 0.07) (**Figures 3C, D** **)**, RER (PLA 0.90 ± 0.01, MET 0.90 ± 0.01, *P* = 0.8), HR (PLA 154 ± 4 bpm, MET 159 ± 4 bpm, *P* = 0.1), blood glucose (PLA 5.1 ± 0.1 mmol/L, MET 4.9 ± 0.1 mmol/L, *P* = 0.2), or blood lactate (PLA 3.2 ± 0.5 mmol/L MET 3.6 ± 0.4 mmol/L, *P* = 0.2).

Likewise, no differences between treatment periods were seen in VO_2_peak (PLA 3.5 ± 0.1, MET 3.5 ± 0.1, *P* = 0.5).

## Discussion

The main finding of this study is that self-selected exercise intensity was not reduced by metformin treatment, despite the fact that metformin treatment significantly increased RPE during the exercise bout with fixed intensity. Instead, we found a tendency towards a higher self-selected exercise intensity with metformin treatment (*P* = 0.09).

There are several potential explanations for this unexpected and apparently contradictory result. An obvious speculation is that self-selected exercise intensity is not affected by RPE. In the literature, however, it is well established that RPE and work intensity are linearly and closely associated (15–17).

Another potential explanation for the findings could be that the RPE assessment was inaccurate and/or imprecise. In this context, Morgan et al. demonstrated that RPE has a considerable intra-individual day-to-day variation (18). In continuation of this, RPE is a subjective variable, which may be affected by a variety of factors. As such, various psychological traits may play a role in the perceptual procession of information related to muscular work and thereby RPE (18).

Taking this into account, it may be speculated that the exercise bout with self-selected intensity, where the participants were asked to keep the exercise intensity corresponding to 14 to 15 on the Borg scale, resulted in large noise-to-signal in the oxygen consumption rate. This could be the case, regardless of the great effort that was put into standardizing the exercise bouts to reduce the impact of confounding factors, potentially affecting RPE. The same problem might have been expected in a reverted version during the exercise bout with fixed intensity, but here it may be argued that the RPE was less influenced by external factors, since RPE was assessed after the exercise bout, in resting conditions, which gave the participants time to carefully consider their answer. Nonetheless, the RPE results must be interpreted cautiously. In continuation of this and despite causality has been debated, RPE and lactate have been shown to be linearly associated (19), and metformin-induced increases in RPE and blood lactate have been reported to be associated (20). In the present study, during the exercise bout with fixed intensity, a tendency towards higher blood lactate was seen in MET compared to PLA (*P* = 0.06), which may support differences in RPE between MET and PLA in the fixed-intensity exercise bout. However, the fact that numerical differences in lactate (and HR) between MET and PLA were comparable between Experimental day A and B speaks against this association between lactate (and HR) and RPE.

When working with a subjective variable such as RPE, another important speculation is whether the order of the treatment periods might have affected the outcomes. To circumvent this, the study was counterbalanced. Moreover, a two-way (treatment*treatment order) repeated-measures ANOVA demonstrated that the treatment order had not influenced the primary outcome (*P* = 0.2 for interaction). Hence, we do not believe that this affected the interpretation of the findings.

Finally, it may be questioned whether a statistically significant difference of 0.8 RPE points on the 6–20 Borg scale is too low to have any clinical relevance, including an effect on self-selected exercise intensity. In this context, it is important that the effects found of metformin on RPE in the current study are in line with previous observations (10, 13). Furthermore, the fact that not only “no difference”, but a tendency towards a higher self-selected exercise intensity with metformin treatment was observed, makes the results convincing, and suggests that clinicians can prescribe metformin without worrying whether self-selected exercise intensity may be negatively affected.

It has been suggested that metformin treatment may reduce mitochondrial respiration and therefore cardiorespiratory fitness *per se* (21), although data are conflicting (22). In this situation, the increased RPE in MET during exercise with fixed intensity could potentially be explained by a lower cardiorespiratory fitness level. However, based on the results from the present study, in which no difference was observed in VO_2_peak between treatments, nothing points towards inhibition of mitochondrial respiration to be the explanation for the increased RPE.

### Limitations

A potential limitation is that the study was performed in healthy individuals instead of patients with type 2 diabetes. However, to our knowledge, there are no indications that the effect of metformin on RPE, lactate and HR should be different in patients with type 2 diabetes than in healthy individuals (23). Nonetheless, if patients with type 2 diabetes had been included, a larger difference in blood lactate between treatments might have been observed, since patients with type 2 diabetes typically have higher blood lactate levels than non-diabetic individuals (24), and since metformin inhibits lactate uptake by the liver. Following this, it may be speculated that more robust differences in blood lactate between treatments would have influenced self-selected exercise intensity.

Another limitation of the present study is the small number of participants, which may lead to both type 1 and type 2 statistical errors. Moreover, the inclusion of only males limits the external validity of the study results.

### Conclusion

In conclusion, this study has shown that RPE is increased by metformin treatment but that this does not lead to lower self-selected exercise intensity in male subjects with normal glucose tolerance. Thus, the clinical importance of the increased RPE during exercise seen with metformin treatment remains unclear.

## Data Availability Statement

The raw data supporting the conclusions of this article will be made available by the authors, without undue reservation.

## Ethics Statement

The studies involving human participants were reviewed and approved by the ethical committee of the capital region of Denmark. The patients/participants provided their written informed consent to participate in this study.

## Author Contributions

NP and KK wrote the manuscript. NP and KK performed the statistical analyses. KK and KH designed the study and conceptualized and designed the analyses with contributions from NP and BP. NP and BP obtained the funding. NP, MJ, NH, and CP-B contributed to data collection, data analysis/processing, and/or data quality control procedures. All authors contributed to drafting the article and/or revising it critically for important intellectual content. All authors approved the final version of the manuscript. All authors accept responsibility for all aspects of the work insofar as ensuring that questions related to the accuracy or integrity of any part of the article are appropriately investigated and resolved. KK is responsible for the integrity of the work as a whole. All authors contributed to the article and approved the submitted version.

## Funding

This work was partially supported by a grant from Aase and Ejnar Danielsen Foundation (NP), a grant from the Research Foundation of Rigshospitalet (E-23606-03) (NP), and Christian d. X Foundation (NP). The Centre for Physical Activity Research is supported by grants from TrygFonden (grants ID 101390 and ID 20045) (BP).

## Conflict of Interest

The authors declare that the research was conducted in the absence of any commercial or financial relationships that could be construed as a potential conflict of interest.
