# Supplementary material for: The Effect of Metformin on Self-Selected Exercise Intensity in Healthy, Lean Males: A Randomized, Crossover, Counterbalanced Trial
Source: Front Endocrinol (Lausanne). 2021 Feb 25;12:599164. doi: 10.3389/fendo.2021.599164 (PMC7947847; doi:10.3389/fendo.2021.599164)
Supplement: Supplementary file 1 [file DataSheet_1.pdf]

Study protocol

TITLE

The effects of metformin on self-selected exercise intensity, physical fitness and exercise-induced AMPK-activation.

BACKGROUND

Physical activity is a first line treatment for patients with type 2 diabetes (T2D), and the effects of physical activity on glycemic control and cardiovascular risk factors in patients with T2D are well-documented (1;2). However, the vast majority of patients with T2D do not achieve satisfying glycemic control with physical activity and other lifestyle interventions alone, which is why pharmacological treatment is most often initiated.
Metformin is the initial glucose-lowering drug of choice for T2D (3;4), and most patients with T2D do receive metformin from shortly after having the T2D diagnosis and thereafter the rest of their life (5). Metformin is a well-tested and, for most patients, well-tolerated drug, which has proven valuable for improving glycemic control and reducing cardiovascular complications (6;7).
Metformin has pleiotropic effects and mechanisms of action, of which the inhibition of Complex I in mitochondria is central (8-10). This in turn leads to increased blood lactate levels (11) and increased 5' adenosine monophosphate-activated protein kinase (AMPK)-activation (12), also in skeletal muscle (13). These mechanisms are analogue to some of the mechanisms by which exercise exerts it's effects, and so an interaction between metformin and exercise is plausible (14). In this context, it has been shown that subjects on metformin, who undergo a training intervention, have inferior improvements in insulin sensitivity and other cardiovascular risk factors, compared to subjects who undergo a similar training intervention without being on metformin (15;16). Thus, it seems that metformin treatment directly reduces the positive effects of physical activity.
Subjects with T2D have lower physical fitness levels compared to healthy controls (17;18), and do typically perform free-living, unsupervised exercise at lower intensities compared to matched, healthy subjects – intensities that are too low to induce metabolic improvements (19;20). The reason for this is unclear, but it is reasonable to believe that the increased rate of perceived exertion (RPE) and increased lactate levels seen during a standardized exercise bout in subjects with T2D compared to healthy controls, plays a role (21). Since metformin treatment results in higher blood lactate levels and RPE for a given exercise intensity (22;23), it may be speculated that metformin treatment is responsible for some kind of 'exercise intolerance'. In continuation of this, and

although causality between lactate levels and RPE is questioned (24), it may be speculated that self-selected exercise intensity will be lower, if subjects are on metformin treatment. Finally, the abdominal discomfort commonly seen with metformin treatment, may also result in lower self-selected exercise intensity. Thus, metformin treatment may indirectly reduce the positive effects of physical activity.

Overall, both direct and indirect mechanisms may in this way be responsible for the potentially reduced effects of physical activity when performed in combination with metformin treatment.

HYPOTHESIS

Metformin treatment reduces self-selected exercise intensity, which may be explained via decreased mitochondrial Complex I function, increased blood lactate levels, heart rate and RPE during exercise. Furthermore, exercise-induced AMPK-activation is reduced with metformin treatment.

AIMS

Primary outcome:

1) To assess the impact of metformin on self-selected exercise intensity.

Secondary outcomes:

1) To assess the impact of metformin on RPE (25), blood lactate, heart rate, oxygen consumption, respiratory exchange ratio and AMPK-activation in skeletal muscle during an exercise bout with fixed intensity.
2) To assess the impact of metformin on physical fitness (VO_2max) and oxidative phosphorylation (in skeletal muscle fibers).

EXPERIMENTAL DESIGN

Sample Size: No studies have, to our knowledge, investigated the effects of metformin on voluntary exercise intensity. The impact of short-term metformin treatment on RPE, blood lactate, heart rate and VO_2max has been investigated in various studies (22;23;26-29). These studies have each included between 9 and 17 subjects. In general, metformin treatment has been found to increase RPE, blood lactate, and heart rate robustly, whereas the effects on VO_2max are less clear.

Based on these studies, and from a pragmatic view, 15 subjects will be included in the study.

Recruitment, information and informed consent: Potential subjects are recruited through direct contact, advertising on the internet and advertising in local newspapers. Potential subjects contact one of the project participants by telephone and are orally informed about the study. If no exclusion

criteria are identified in the phone, an information meeting date is arranged, and the potential subject is informed about the possibility of inviting a private counselor to the information meeting. Prior to the information meeting, written project information including consent form will be sent to the potential subject and he/she will be encouraged to read the information thoroughly.
At the information day, extensive oral information about the scientific study will be given by one of the scientific participants. The information will be given in a closed room where only the scientific participant, the potential subject and the potential private counselor will be present. After the oral information has been given, the potential subject will be asked if he/she is ready to decide whether or not to participate in the study. If the potential subject is ready and wants to participate in the study, the informed consent form will be signed and the screening will commence. If the potential subject wants' additional time to consider whether or not to participate in the study, an agreement about a telephone meeting after approx. one week is made. The subject will be informed of the possibility of contacting one of the scientific participants by telephone, in case of questions. If the potential subject wants to participate in the study, a screening will be arranged and the signed consent form will be brought to the screening by the subject.
Subjects and screening: A total of n=15 healthy, lean (BMI<25), low-to-moderately physically active (≤ 150 min of structured physical activity/week), male subjects will be included in the study. A medical examination, medical history, blood chemistry screen, ECG, oral glucose tolerance test (OGTT), Dual x-ray absorptiometry (DXA) scan and a physical fitness test (VO_2max test) will be performed. If an individual meets the inclusion criteria, informed oral and written consent will be obtained prior to participation in the study, in accordance with guidelines developed by the ethical committees.

Inclusion Criterias:

- Male
- Normal glucose tolerance (HbA1c < 39 mmol/mol)
- BMI < 25
- Structured physical activity ≤ 150 min/week
- Apparently healthy

Exclusion Criterias:

- Smoking
- Daily pharmaceutical treatment
- Contraindication to increased levels of physical activity (30)

- Liver cell damage (ALT/AST elevated 3 times above upper normal values)
- Renal insufficiency (eGFR<60 ml/min)
- Prior history of lactic acidosis

STUDY PLAN

Subjects will be included in a double-blinded, cross-over study with 2 trials, each consisting of two experimental days. Trials will be performed in a randomized order. Trials will be identical besides the following treatment:

1) Metformin treatment (17 days)
2) Placebo treatment (17 days)

To avoid low adherence to the treatment protocols due to gastrointestinal discomfort, treatment will be up-titrated in the following way:

- Treatment Day 1-4: 500 mg x 2
- Treatment Day 5-8: 500 + 1000 mg
- Treatment Day 9-17: 1000 mg x 2

Since metformin commonly induces abdominal discomfort, which may influence dietary intake, subjects will be asked to keep diet records from Day 13-16 during the first trial. At Day 13-16 in the second trial, subjects will be given a copy of their diet record and instructed to follow this closely. Moreover, subjects will be instructed not to perform any vigorous physical activity from Day 13 and onwards in both trials.

The Experimental Days (1 and 2) will be performed at Treatment Day 15 and 17, respectively, and will consist of the following:

Experimental Day 1:

- T = -120 min: Small, standardized breakfast at home (with morning dose of metformin)
- T = -45 min: Arriving in lab, acclimatization
- T = 0 min: Blood sample, start of exercise bout with self-selected exercise intensity
- T = 15+30 min: Blood samples, RPE
- T = 45 min: Blood sample, end of exercise intervention, RPE
- T = 60 min: Mixed Meal Tolerance Test (MMTT) with blood sampling every 30 min
- T = 180 min: VO_2max test

Experimental Day 2:

- T = -120 min: Small, standardized breakfast at home (with morning dose of metformin)
- T = -45 min: Arriving in lab, acclimatization
- T = -15 min: Muscle biopsy
- T = 0 min: Blood sample, start of exercise bout with fixed exercise intensity
- T = 15 + 30 min: Blood sample, RPE
- T = 45 min: End of exercise intervention, muscle biopsy, blood sample, RPE

METHODS

Exercise bouts: The exercise bouts will be performed at a cycle ergometer (Monarc 739E, Varberg, Sweden). At the bout with self-selected exercise intensity, subjects will be encouraged to keep the RPE during exercise equal to 14 on the Borg scale (between 'Somewhat Hard' and 'Hard'). The subject will be encouraged to adjust the load to meet the required RPE continuously, and will be blinded to the load.

At the bout with fixed intensity, subjects will exercise at 70% of VO_2max (based on the VO_2max-test performed on the screening day). This intensity has previously been shown to be sufficient to activate AMPK (13;31;32). RPE will be assessed during and after the exercise bout. Indirect calorimetry (CPET, Cosmed, Italy) and heart rate (Polar RS400, Kempele, Finland) measurements will be performed continuously during the exercise interventions using a mask + breath-by-breath measurements and heart rate strap, respectively.

Blood samples will be taken in heparinized syringes before, during and after the exercise bouts and immediately analyzed for glucose and lactate (ABL 7 series, Radiometer, Denmark). Moreover, blood samples for subsequent analyses of catecholamines will be collected.

Muscle biopsies: Muscle biopsies will be taken prior to and immediately after the exercise bout with fixed intensity. Under local anaesthesia (lidocain 2%, 3-5 ml) a muscle biopsy will be obtained from vastus lateralis of the quadriceps femoris muscle. Muscle tissue will be separated in two portions: On portion will be immediately frozen in liquid nitrogen and stored at -80°C until analysis. Analyses will consist of AMPK/ACC phosphorylation, AMPK activity (33) and AS160 phosphorylation. Another portion will be used to measure oxidative phosphorylation of permeabilized single muscle fibers (34).

MMTT: A liquid MMTT (450 kcal with a macronutrient composition that resembles a normal meal; 55% carbohydrates, 30% fat and 15% protein will commence 15 min after each intervention. Blood samples will be taken for glucose, insulin and C-peptide measurements.

VO_2max-test: A standard VO_2max test will be performed at a cycle ergometer (Monarc 739E) using indirect calorimetry (CPET). After a 5 min warm-up at 75W, load will be increased by 25W every minute until exhaustion.

RISKS, ADVERSE EFFECTS AND DISCOMFORT

VO_2-max test: A physical fitness test, where subjects must put in maximum effort. This will cause some degree of breathlessness. VO_2max test is a standard method used for scientific purposes in our laboratory.

DXA scan: Is not expected to cause significant discomfort. The radiation acquired is so small that it doesn't cause any risk to subjects. DXA-scan is a standard method used for scientific purposes in our laboratory.

Metformin treatment: Common side effects of metformin are abdominal discomfort and diarrhea (up to 50%) (35). In healthy subjects, single and multiple doses of metformin showed no effect on plasma glucose (36). Lactic acidosis is a rare, but serious, metabolic complication that can occur due to metformin accumulation during treatment with metformin tablets. The reported incidence of lactic acidosis in patients receiving metformin is very low (approximately 0.03 cases/1000 patient-years, with approximately 0.015 fatal cases/1000 patient-years). In more than 20,000 patient-years exposure to metformin in clinical trials, there were no reports of lactic acidosis (37).

Blood sampling: Will cause minor discomfort in terms of a venous catheter. There is theoretically a risk for infections introduced through the catheter. The blood volume collected is so small that it will cause no symptoms.

Muscle biopsies: The injection of local anaesthetics may cause a transient burning pain in the skin. Bleeding and infections are rarely seen. The actual execution of the biopsy may cause discomfort and pain. Following the biopsy, there is often a localized tenderness for 1-3 days. In some cases there may be a greater bruising around the injection site, resulting in a stronger tenderness, which may last several days. Furthermore, when making the incision, a nerve in the skin can be cut. This only happens rarely and can cause a small area of the skin to become numb. In most cases, the sensory perception of the skin will return gradually and be normalized after a few months. After the biopsy, a small scar of about 8-10 mm will be visible.

SUBJECT'S PHYSICAL AND MENTAL INTEGRITY AND PRIVACY

The study will be reported to "Datatilsynet" through Rigshospitalets joint review.

The "Lov om behandling af personoplysninger" will be respected.

ECONOMICAL SUPPORT

Kristian Karstoft and Katrine B. Hansen have initiated the study. Different private foundations will be applied for economical support (see attached budget). Centre for Physical Activity Research will cover the costs not covered by external foundations. No commercial institutions have been or will be applied for economical support. None of the scientific participants have any disclosures to declare.

Subjects will be paid a fee of DKK 5,000 for participation in the study. The fee is taxable. The fee covers discomfort and travel expenses. The fee is paid by the end of the subject's participation. If the subject is excluded or choose to withdraw from the trial, a fee equal to the part of the study the subject has completed will be paid.

ETHICAL ASPECTS

The project is, as described above, expected to result in limited risks, adverse effects and discomfort to the subjects. The subjects will benefit from the study in terms of a thorough medical examination. For research in general, the study is sound and important, and it will contribute to our knowledge about how to physical activity and metformin treatment – two cornerstones in the treatment of T2D - may interact, something which may potentially have a huge impact in future treatment of and recommendations for subjects with T2D.

STATISTICAL ANALYSES

All data will be tested for normality and homogeneity of variance using Kolmogorov-Smirnov and Levene tests. Variables that diverge from parametric assumptions will be log-transformed prior to analysis. Between-intervention variables will be compared via Student’s paired t-test and two-way repeated-measure analysis of variance (RM-ANOVA). Relationships between changes in variables will be examined using regression analyses.

BIOLOGICAL MATERIAL

Blood and muscle biopsies will be collected in the study. This will be used to assess the potential differential changes in variables following the interventions.

Approx. 250 mL blood will be collected during the entire study. Four muscle biopsies will be taken in total, all from m. quadriceps femoris, vastus lateralis. Each biopsy is approx. 100-200 mg.

Primary muscle cell cultures will be established from muscle biopsies.

The biological material will be stored in a research biobank for a maximum of 20 years and after that, it will be destroyed. If any later studies want to make use of the biological material, this will

only take place following approval by the ethical committee.
The biological material will not leave Denmark.

EXPECTED OUTCOMES

It is expected that this project will show that metformin treatment results in lower self-selected exercise intensity and lower AMPK-activation of exercise with fixed intensity. This will add important information to the potential benefits or drawbacks of the metformin-exercise dual treatment, which today is standard for all T2D patients.

DISSEMINATION

At least one manuscript will be produced from the data and published in an international peer-reviewed journal. Positive, negative and inconclusive results will be published.

STUDY LOCATION

Centre for Physical Activity Research (CFAS)
Rigshospitalet
Tagensvej 20, section M7641
DK-2100 Copenhagen
Denmark

SCIENTIFIC PARTICIPANTS

Kristian Karstoft, MD, PhD, CFAS, Rigshospitalet
Katrine Bagge Hansen, MD, PhD, CFAS, Rigshospitalet
Nanna Skytt Pilmark, MD, CFAS, Rigshospitalet
Christina Petersen-Bønding, Stud. MSc, CFAS, Rigshospitalet
Niels Frederich Holm, Stud MD, CFAS, Rigshospitalet
Helga Ellingsgaard, PhD, CFAS, Rigshospitalet
Jonas Møller Kristensen, PhD, August Krogh Institute, University of Copenhagen
Jens Frey Halling, MSc, Stud PhD, Biological Institute, University of Copenhagen
Henriette Pilegaard, PhD, Professor, Biological Institute, University of Copenhagen
Marc Y Donath, MD, PhD, Professor, Basel Universitätsspital, Switzerland
Bente Klarlund Pedersen, MD, DMSc, professor, CFAS, Rigshospitalet

Reference List

(1) Snowling NJ, Hopkins WG. Effects of different modes of exercise training on glucose control and risk factors for complications in type 2 diabetic patients: a meta-analysis. Diabetes Care 2006 Nov;29(11):2518-27.

(2) Boule NG, Haddad E, Kenny GP, Wells GA, Sigal RJ. Effects of exercise on glycemic control and body mass in type 2 diabetes mellitus: a meta-analysis of controlled clinical trials. JAMA 2001 Sep 12;286(10):1218-27.

(3) Standards of medical care in diabetes--2014. Diabetes Care 2014 Jan;37 Suppl 1:S14-S80.

(4) Ryden L, Grant PJ, Anker SD, Berne C, Cosentino F, Danchin N, et al. ESC Guidelines on diabetes, pre-diabetes, and cardiovascular diseases developed in collaboration with the EASD: the Task Force on diabetes, pre-diabetes, and cardiovascular diseases of the European Society of Cardiology (ESC) and developed in collaboration with the European Association for the Study of Diabetes (EASD). Eur Heart J 2013 Oct;34(39):3035-87.

(5) Mor A, Berencsi K, Svensson E, Rungby J, Nielsen JS, Friborg S, et al. Prescribing practices and clinical predictors of glucose-lowering therapy within the first year in people with newly diagnosed Type 2 diabetes. Diabet Med 2015 Dec;32(12):1546-54.

(6) Effect of intensive blood-glucose control with metformin on complications in overweight patients with type 2 diabetes (UKPDS 34). UK Prospective Diabetes Study (UKPDS) Group. Lancet 1998 Sep 12;352(9131):854-65.

(7) Stratton IM, Adler AI, Neil HA, Matthews DR, Manley SE, Cull CA, et al. Association of glycaemia with macrovascular and microvascular complications of type 2 diabetes (UKPDS 35): prospective observational study. BMJ 2000 Aug 12;321(7258):405-12.

(8) Owen MR, Doran E, Halestrap AP. Evidence that metformin exerts its anti-diabetic effects through inhibition of complex 1 of the mitochondrial respiratory chain. Biochem J 2000 Jun 15;348 Pt 3:607-14.

(9) Bridges HR, Jones AJ, Pollak MN, Hirst J. Effects of metformin and other biguanides on oxidative phosphorylation in mitochondria. Biochem J 2014 Sep 15;462(3):475-87.

(10) Wessels B, Ciapaite J, van den Broek NM, Nicolay K, Prompers JJ. Metformin impairs mitochondrial function in skeletal muscle of both lean and diabetic rats in a dose-dependent manner. PLoS One 2014;9(6):e100525.

(11) Fery F, Plat L, Balasse EO. Effects of metformin on the pathways of glucose utilization after oral glucose in non-insulin-dependent diabetes mellitus patients. Metabolism 1997 Feb;46(2):227-33.

(12) Hawley SA, Ross FA, Chevtzoff C, Green KA, Evans A, Fogarty S, et al. Use of cells expressing gamma subunit variants to identify diverse mechanisms of AMPK activation. Cell Metab 2010 Jun 9;11(6):554-65.

(13) Musi N, Fujii N, Hirshman MF, Ekberg I, Froberg S, Ljungqvist O, et al. AMP-activated protein kinase (AMPK) is activated in muscle of subjects with type 2 diabetes during exercise. Diabetes 2001 May;50(5):921-7.

(14) Malin SK, Braun B. Impact of Metformin on Exercise-Induced Metabolic Adaptations to Lower Type 2 Diabetes Risk. Exerc Sport Sci Rev 2016 Jan;44(1):4-11.

(15) Malin SK, Gerber R, Chipkin SR, Braun B. Independent and combined effects of exercise training and metformin on insulin sensitivity in individuals with prediabetes. Diabetes Care 2012 Jan;35(1):131-6.

(16) Malin SK, Nightingale J, Choi SE, Chipkin SR, Braun B. Metformin modifies the exercise training effects on risk factors for cardiovascular disease in impaired glucose tolerant adults. Obesity (Silver Spring) 2013 Jan;21(1):93-100.

(17) Regensteiner JG, Sippel J, McFarling ET, Wolfel EE, Hiatt WR. Effects of non-insulin-dependent diabetes on oxygen consumption during treadmill exercise. Med Sci Sports Exerc 1995 May;27(5):661-7.

(18) Regensteiner JG, Sippel J, McFarling ET, Wolfel EE, Hiatt WR. Effects of non-insulin-dependent diabetes on oxygen consumption during treadmill exercise. Med Sci Sports Exerc 1995 Jun;27(6):875-81.

(19) Johnson ST, Tudor-Locke C, McCargar LJ, Bell RC. Measuring habitual walking speed of people with type 2 diabetes: are they meeting recommendations? Diabetes Care 2005 Jun;28(6):1503-4.

(20) Tudor-Locke C, Bell RC, Myers AM, Harris SB, Ecclestone NA, Lauzon N, et al. Controlled outcome evaluation of the First Step Program: a daily physical activity intervention for individuals with type II diabetes. Int J Obes Relat Metab Disord 2004 Jan;28(1):113-9.

(21) Kim YS, Seifert T, Brassard P, Rasmussen P, Vaag A, Nielsen HB, et al. Impaired cerebral blood flow and oxygenation during exercise in type 2 diabetic patients. Physiol Rep 2015 Jun;3(6).

(22) Boule NG, Robert C, Bell GJ, Johnson ST, Bell RC, Lewanczuk RZ, et al. Metformin and exercise in type 2 diabetes: examining treatment modality interactions. Diabetes Care 2011 Jul;34(7):1469-74.

(23) Malin SK, Stephens BR, Sharoff CG, Hagobian TA, Chipkin SR, Braun B. Metformin's effect on exercise and postexercise substrate oxidation. Int J Sport Nutr Exerc Metab 2010 Feb;20(1):63-71.

(24) Hall MM, Rajasekaran S, Thomsen TW, Peterson AR. Lactate: Friend or Foe. PM R 2016 Mar;8(3 Suppl):S8-S15.

(25) Borg GA. Psychophysical bases of perceived exertion. Med Sci Sports Exerc 1982;14(5):377-81.

(26) Braun B, Eze P, Stephens BR, Hagobian TA, Sharoff CG, Chipkin SR, et al. Impact of metformin on peak aerobic capacity. Appl Physiol Nutr Metab 2008 Feb;33(1):61-7.

(27) Johnson ST, Robert C, Bell GJ, Bell RC, Lewanczuk RZ, Boule NG. Acute effect of metformin on exercise capacity in active males. Diabetes Obes Metab 2008 Sep;10(9):747-54.

(28) Learsi SK, Bastos-Silva VJ, Lima-Silva AE, Bertuzzi R, De Araujo GG. Metformin improves performance in high-intensity exercise, but not anaerobic capacity in healthy male subjects. Clin Exp Pharmacol Physiol 2015 Oct;42(10):1025-9.

(29) Sharoff CG, Hagobian TA, Malin SK, Chipkin SR, Yu H, Hirshman MF, et al. Combining short-term metformin treatment and one bout of exercise does not increase insulin action in insulin-resistant individuals. Am J Physiol Endocrinol Metab 2010 Apr;298(4):E815-E823.

(30) Pedersen BK, Saltin B. Exercise as medicine - evidence for prescribing exercise as therapy in 26 different chronic diseases. Scand J Med Sci Sports 2015 Dec;25 Suppl 3:1-72.

(31) Bartlett JD, Hwa JC, Jeong TS, Louhelainen J, Cochran AJ, Gibala MJ, et al. Matched work high-intensity interval and continuous running induce similar increases in PGC-1alpha mRNA, AMPK, p38, and p53 phosphorylation in human skeletal muscle. J Appl Physiol 2012 Apr;112(7):1135-43.

(32) Kjobsted R, Pedersen AJ, Hingst JR, Sabaratnam R, Birk JB, Kristensen JM, et al. Intact Regulation of the AMPK Signaling Network in Response to Exercise and Insulin in Skeletal Muscle of Male Patients With Type 2 Diabetes: Illumination of AMPK Activation in Recovery From Exercise. Diabetes 2016 May;65(5):1219-30.

(33) Birk JB, Wojtaszewski JF. Predominant alpha2/beta2/gamma3 AMPK activation during exercise in human skeletal muscle. J Physiol 2006 Dec 15;577(Pt 3):1021-32.

(34) Boushel R, Gnaiger E, Schjerling P, Skovbro M, Kraunsoe R, Dela F. Patients with type 2 diabetes have normal mitochondrial function in skeletal muscle. Diabetologia 2007 Apr;50(4):790-6.

(35) "Product Information. Glucophage (metformin)." Bristol-Myers Squibb, Princeton, NJ. 2016.
Ref Type: Generic

(36) Sambol NC, Chiang J, O'Conner M, Liu CY, Lin ET, Goodman AM, et al. Pharmacokinetics and pharmacodynamics of metformin in healthy subjects and patients with noninsulin-dependent diabetes mellitus. J Clin Pharmacol 1996 Nov;36(11):1012-21.

(37) FDA: Drugs.com. 2016.
Ref Type: Generic
